# Report of the County Medical Officer of Health

**Published:** 1919-09-13

**Authors:** 


### LONDON AND THE INFLUENZA OUTBREAKS.

### Report of the County Medical Officer of Health.

FOLLOWING on the many discussions and supposed discoveries in connection with the recent appearances of this disease, the report issued by Dr. W. H. Hamer makes most interesting reading to those who, in the midst of much apparently useless argument and most unfavourable local conditions, had to grapple with case after case of acute infection. At the outset, attention is drawn to the close relation between the appearance of cerebro-spinal fever, acute poliomyelitis and influenza as seen in the years 1914-17, and in this connection we would also refer our readers to the Chadwick lectures delivered by Dr. F. G. Crookshank. But, true to its reputation, influenza has again created the impression of a "new disease" and in spite of the many prophecies and surmises made by authorities prior to 1918, realisation has in many particulars differed from anticipation. In considering the conditions particularly in London, there is at least the advantage that in the past more has been written about influenza in London than anywhere else in the present review, and considerable assistance is obtained by comparison with the old-time records. We may quote from Dr. Hamer's introduction to illustrate his meaning:

> For adequate treatment of the subject an "extensive and peculiar" knowledge, at least of the London influenza, is to be desired; but any contributions to this will be of little value if the subject be not approached from the natural history point of view. As Creighton has said in another connection, the older or Hippocratic method must be applied. This is one which took account of gradations, modifications, and affinities, being careless of symmetry, of definitions, or clear-cut nosological ideas, or the dividing lines of classifications.

We have lately read many detailed studies of the influenzal group of diseases from this standpoint and, in the report, as in this short reference, it has been impossible to indicate more than their general trend. Here the subject has been considered under two main heads, namely, a review of the recent influenzal prevalences and a study of the light thrown upon these epidemics by earlier prevalences, with a summary view of the epidemiology of the influenzal group.

### RECENT PREVALENCE.

Referring to the weekly registered deaths from influenza, pneumonia, and bronchitis for the whole of 1918 and the first nineteen weeks of 1919 which are set out in tabular form, one notes that after the first indication of the re-development afforded by a rise in the general death-rate, and in the rates for respiratory diseases in the 51st week of 1917, the maximum number of deaths from bronchitis (283) and influenza was attained in the second week of 1918, and marked the first crest of the waves of increased mortality which followed later. At this period it was noteworthy that there were a large number of mild influenzal cases in London, and the epidemic continued to smoulder until a second smaller but still definite wave occurred between the ninth and eleventh weeks.

The mortality was not, in fact, alarming, but its importance lies in the first recording of the "obscure disease with cerebral symptoms." Very rapidly the suspicion became confirmed that there was something new and special in the behaviour of influenza in London. A number of institutional outbreaks had appeared in both England and Scotland. In many of these a special epidemic influence was confirmed by the occurrence of some dozens of cases presenting unusual cerebral symptoms, and in spite of the original explanation of "botulism" provided, full investigation resulted in the issue, in May, of a memorandum stating that the earliest traceable case of the "obscure disease with cerebral symptoms," as it was agreed to call it provisionally, developed on January 26, but that there was no particular food found to be related to the outbreak, and no bacteriological evidence of true botulism. Cases continued to appear, but by June, when the botulism theory had been finally discredited, there was fairly good agreement among the profession that what was strictly "new" was the occurrence in epidemic form of cases of *encephalitis*, not necessarily due to the action of a known infective agent, but presenting clinical symptoms, any or all of which can be noted in the various kinds of encephalitis with which the neurologists have long been conversant.

### FROM JUNE 1918 ONWARDS.

There were then three distinct epidemics to be considered. The first or summer outbreak produced an increase in the general death-rate for London in the week ending June 22. The increase was limited mainly to the South-Eastern district, and in the light of sickness rates among adults, the epidemic appears to have reached its maximum about the end of the first week in July, when deaths were nearly 400, and to have practically ended by the middle of August. By the time, however, that deaths from influenza again fell to normal, there were abundant signs of a fresh outbreak, and the autumn epidemic began in the early part of October. In the last week of that month nearly 3,000 deaths were registered from influenza and pneumonia, but in spite of the great severity, the period between the onset and the crisis was about the same as before, namely, between three and four weeks. School attendances were markedly affected until the end of the autumn session, and deaths from influenza remained high until the end of the year. The third outbreak began in the latter part of January 1919. but was of a less severe character, and children of school age were noticeably less affected during this third prevalence.

**London and the Influenza Outbreaks—(*continued*).**

The Severity of the Epidemics.

Two views must be taken: the number of cases and the number of deaths. For the first it is to be noted that both the fall in school attendance and the adult sickness rates were very much greater in the autumn epidemic. These figures are compared in relation to deaths in the two classes, and it is seen that while clinical cases of influenza in the autumn were about twice as numerous as in the summer, the deaths, registered from or attributable to epidemic influenza were more than ten times, and the case mortality about five times as heavy. The deaths in London certified to be due to influenza for the middle of June 1918 to May 3, 1919, numbered 16,520, and of these about 989 may be allocated to the summer, 11,898 to the autumn, and 3,633 to the winter epidemic.

Age Incidence in 1918.

The age incidence of influenza mortality in 1918 offers a remarkable contrast to the experience of the past when the characteristic has usually been a high incidence in infancy and old age.

There was observable in the epidemic of 1890, the first of any importance after that of 1847-8, a relatively higher incidence at ages 20-45 than in 1847-8 or in the succeeding years 1891-2; indeed, there was a progressive decline in the relative mortality at these ages in 1891 and 1892 upon that of 1890; and a corresponding increase in incidence at higher ages. In 1893, however, age incidence reverts to that of 1890; and from a comparison of the change of age incidence in the six years 1890-95 the only definite conclusion to be drawn is that the greater the number of deaths, the smaller the proportion at ages 20-45. With this rule the 1918 deaths are directly at variance. Nor is it easy to frame any theory of immunity acquired in the epidemics of 1890-95, which fully account for the abnormal age incidence of 1918.

Dr. Hamer makes, however, a number of highly interesting speculations on the causes of this anomaly, and also deals extensively with the effects produced upon the children of elementary schools. Incidence in relation to exposure to infection is also discussed, but owing to obvious circumstantial difficulties there is little definite information to be gleaned in this direction. Meteorological conditions are reviewed, but, as the author states, "no identical conditions of excess or deficiency in the principal meteorological elements appear to mark the periods of the epidemic."

Origin of the Outbreaks.

The veil which the war has drawn over so many European happenings makes the tracing of disease movements not a little difficult. Apparently the Spanish origin for the infection is completely refuted. In April, the American troops were definitely infected and relationship seems to be established between the European epidemic and an abnormal prevalence of influenza and pneumonia in New York which occurred in March.

The absence of any marked increase in the death-rates of New York and Chicago, between April and the advent of the autumn pandemic, certainly suggests that this spring prevalence (the usual mild grippe-like disease of American cities) was the American equivalent of the European epidemic. It may be added that the weekly bulletin of the New York Department of Health refers to the weather experienced during January and February 1918 as "two months of the most severe weather which this city has ever experienced."

In spite of many handicaps encountered, attempt is made to trace the world course of many of the infection waves, and the relation of London to them. Even though the description is lacking in parts in detail, yet some valuable and interesting facts are recorded and have particular interest to those who encountered the disease overseas at different periods of the war.

Previous Prevalence of Influenza.

As an introduction to Part II. of the report, Dr. Hamer provides a very concise account of the past history of the disease, beginning with the fifteenth century, but space does not here allow of more than the reference. The conclusions drawn are, however, of the greatest importance. Allowing for variations in nomenclature and reading generally between the lines, there seems to have been increased mortality from both cerebro-spinal fever and poliomyelitis from about five years before to some five years after the great pandemics of influenza. Similarly, some five years prior to the explosion of 1918, there was again a growing diffusion of these two diseases. In thickly populated areas there seems to have been a law of coincidence or overlapping between cerebro-spinal fever, poliomyelitis and influenza. Two other points are also brought out. In the nineteenth century there was a definite tendency to alternate between the Eastern and Western hemispheres, and also the constitutions are seen to extend over more years in the later centuries, suggesting that the density of population, freedom of communication and immunisation by previous attacks play a great part in the disease. On a review of the whole matter, it would seem that the denser the population the more likely is the disease to maintain itself for a series of years about a pandemic prevalence; in the more thinly populated countries prevalences, when they do occur, are likely to be severe ones in contrast to those in large partially immunised centres where the disease may smoulder for years and a great epidemic be only possible a long time after the last pandemic prevalence. Also a valuable comparison may be made with the simple measles curves in London as explained in the Milroy Lectures of 1906.

Having, so to speak, removed susceptible material each "trailer" will spread less quickly, but as one succeeds another, the cerebro-spinal and pulmonary *complications* assume greater apparent prominence, the prevalences are more prolonged and occur at longer intervals until the very existence of influenza in a town like London may be almost forgotten. As the protection of the population wanes, the anomalous illnesses already mentioned again attract attention, encephalitis or a "new" disease with more protean manifestations appears and the town gradually realises that it is again in the grip of an influenza outbreak.

